# Comment on “mt-Keima detects PINK1-PRKN mitophagy in vivo with greater sensitivity than *mito*-QC”

**DOI:** 10.1080/15548627.2021.1907269

**Published:** 2021-04-05

**Authors:** Ian G. Ganley, Alexander J. Whitworth, Thomas G. McWilliams

**Affiliations:** a MRC Protein Phosphorylation & Ubiquitylation Unit, School of Life Sciences, University of Dundee, Dundee, Scotland, UK; b MRC Mitochondrial Biology Unit, University of Cambridge, Cambridge, UK; c Translational Stem Cell Biology and Metabolism, Research Programs Unit, Faculty of Medicine,University of Helsinki, Helsinki, Finland; d Department of Anatomy, Faculty of Medicine,University of Helsinki, Helsinki, Finland

One aspect of selective autophagy that has garnered intense interest is mitochondrial autophagy (mitophagy), particularly PINK1-PRKN-mediated mitophagy as this has direct implications for Parkinson disease. However, initial studies of different mitophagy reporters in this context have resulted in a perceived conflict of results/outcomes and conclusions. A recent article by Liu *et al*. attempts to reconcile these discordant findings by comparing side-by-side two mitophagy reporter systems, mt-Keima and *mito*-QC [1]. A direct comparison of the reporter systems is certainly warranted, and in the cell-based analyses used in Liu *et al*. that involve PRKN overexpression and flow cytometry, it is encouraging to see that mt-Keima produces a robust signal. However, the headline claim that *mito*-QC is insufficiently sensitive for monitoring PINK1-PRKN-dependent mitophagy should be brought into balance. *mito*-QC has been used in multiple published studies by independent groups as part of a series of experiments to clearly and reliably show increased mitophagy, either in the presence or absence of PINK1-PRKN, and under endogenous or overexpressed PRKN conditions. Readers are encouraged to examine some of the many published, well controlled and validated studies [2–22].

Additionally, Liu *et al*. repeated their own results demonstrating that exhaustive exercise induces PINK1-dependent mitophagy in the heart of mt-Keima mice. This is very encouraging given the difficult reproducibility associated with this type of exhaustive exercise study. However, we would like to point out that comparisons between *mito*-QC and mt-Keima should be made cautiously and are somewhat akin to comparing apples to oranges. One compelling reason is because the models have been generated and maintained using different mouse strains (C57BL/6 j-ntac *versus* FVB/NJ for mt-Keima [12,23]). Compared to C57 lines, FVB mice are naturally hyperactive with circadian dysregulation and behavioral defects [24–26], display neuroanatomical abnormalities [27], retinal neurodegeneration and blindness [28]. It is important to note that significant differences also exist between FVB and C57 lines in adapting to and entrainment for treadmill exercise paradigms [29], including mitochondrial differences in muscle tissues [30] and divergent cardiac physiology [31]. We would like to refer the reader to the article by Enriquez that highlights the importance of considering genetic backgrounds in order to meaningfully compare data [32].

Finally, we would like to address the notion by Liu *et al*. that McWilliams *et al*., 2018 and Lee *et al*., 2018 have generated controversy in terms of the PINK1-PRKN mitophagy pathway [7,13]. From our perspective there is no controversy that multiple mitophagy pathways exist. The main conclusion of these papers was that the PINK1-PRKN pathway did not regulate mitophagy under basal conditions. This is the exact same observation that Liu *et al*. demonstrate in their current study: in the absence of exhaustive exercise, mitophagy levels in heart tissues of mt-Keima mice remain constant regardless of the presence or absence of PINK1; i.e., their basal level of mitophagy is independent of PINK1. We would also like to highlight work from the Goessling Lab that recently generated zebrafish models of mitophagy and compared Keima- and tandem mCherry-GFP-based reporters [33]. Here, the authors noted that both reporters faithfully monitored mitophagy and, additionally, the characterized mitophagy was determined to be independent of PINK1 and PRKN, instead requiring BNIP3. Thus, taking into account multiple publications from independent laboratories, using three popular animal models, we think that all demonstrate the same main conclusion, which is that the PINK1-PRKN pathway does not significantly contribute to basal mitophagy.

We think that both reporters have merit and, under different scenarios, use of one type of reporter may be more advantageous than the other. Due to loss of signal upon fixation, use of the mt-Keima mouse requires very strict time-dependent preparation and analysis, which may not be achievable in some laboratories. This is where use of *mito*-QC may be preferable, particularly when immunohistochemistry is required to identify specific cell types or additional cellular components. Targeting mitophagy is likely to have genuine translational importance, most notably for neurodegenerative disorders, particularly given the 99.7% failure rate for drugs in this area [34]. Accordingly, despite the limitations of both reporter systems, a carefully considered discussion is essential. Both reporter systems have contributed to our understanding of physiological mitophagy and are well-positioned to continue unraveling the mysteries of mitophagy in health and disease.
